# A Survey of Eyespot Sexual Dimorphism across Nymphalid Butterflies

**DOI:** 10.1155/2013/926702

**Published:** 2013-12-05

**Authors:** Christopher K. Tokita, Jeffrey C. Oliver, Antónia Monteiro

**Affiliations:** ^1^Yale College, New Haven, CT 06511, USA; ^2^Department of Ecology and Evolutionary Biology, Yale University, New Haven, CT 06511, USA; ^3^Department of Zoology, Oregon State University, Corvallis, OR 97331, USA; ^4^Department of Biological Sciences, National University of Singapore and Yale-NUS College, Singapore 117543

## Abstract

Differences between sexes of the same species are widespread and are variable in nature. While it is often assumed that males are more ornamented than females, in the nymphalid butterfly genus *Bicyclus*, females have, on average, more eyespot wing color patterns than males. Here we extend these studies by surveying eyespot pattern sexual dimorphism across the Nymphalidae family of butterflies. Eyespot presence or absence was scored from a total of 38 wing compartments for two males and two females of each of 450 nymphalid species belonging to 399 different genera. Differences in eyespot number between sexes of each species were tallied for each wing surface (e.g., dorsal and ventral) of forewings and hindwings. In roughly 44% of the species with eyespots, females had more eyespots than males, in 34%, males had more eyespots than females, and, in the remaining 22% of the species, there was monomorphism in eyespot number. Dorsal and forewing surfaces were less patterned, but proportionally more dimorphic, than ventral and hindwing surfaces, respectively. In addition, wing compartments that frequently displayed eyespots were among the least sexually dimorphic. This survey suggests that dimorphism arises predominantly in “hidden” or “private” surfaces of a butterfly's wing, as previously demonstrated for the genus *Bicyclus.*

## 1. Introduction

Sexual dimorphisms are widespread and variable in nature and can result from either natural or sexual selection acting differentially on male and female traits [1–4]. Often, extensive mate-choice experiments, predation experiments, or documentation of sex-specific life histories need to be performed in single species before we understand which form of selection led to the evolution of the sex-specific traits [2]. A complementary approach to direct experimentation, however, is to survey the patterns of sexual dimorphism across a group of closely related organisms and discover whether these are congruent with the findings obtained for single members of the group. These comparative studies across species can help address whether the experimental knowledge obtained for a few species is generalizable across species.

The eyespots in the nymphalid butterfly species *Bicyclus anynana* have been the subject of multiple laboratory experiments that concluded that they are likely evolving under both natural and sexual selection. Mate choice experiments as well as predation and mark-recapture experiments indicated that the dorsal eyespots are involved in mate signaling, whereas ventral eyespots play a role in deflecting the attacks of vertebrate predators [5–8]. In addition, this species is sexually dimorphic regarding eyespot number with females displaying, on average, one more hindwing dorsal eyespot than males (E. Westerman, pers. comm.).

Comparative studies performed across 54 species in the genus *Bicyclus* indicated that eyespots on the dorsal and forewing surfaces were likely evolving under disruptive sexual selection, whereas eyespots on the ventral and hindwing surfaces were likely evolving under stabilizing natural selection [9]. In addition, patterns of eyespot gains and losses, explored separately across males and females of the genus, showed that sexual dimorphisms were likely the result of both sex-limited gains and sex-limited losses of eyespots, most often leading to females becoming the more ornamented sex [10].

Outside of *Bicyclus*, it is currently unclear whether female-biased eyespot ornamentation is a typical feature of nymphalid butterflies and whether eyespots in particular wing surfaces are especially prone to evolve sexual dimorphism. In order to address these questions, we conducted a survey of eyespot sexual dimorphism across the Nymphalidae. We documented the presence or absence of eyespots across the dorsal and ventral wing surfaces of both males and females in 450 different nymphalid species belonging to 399 different genera. We used these presence/absence data to quantify sexual dimorphism and evaluate how dimorphism was distributed across the wing surfaces.

## 2. Materials and Methods

**Figure 1:**
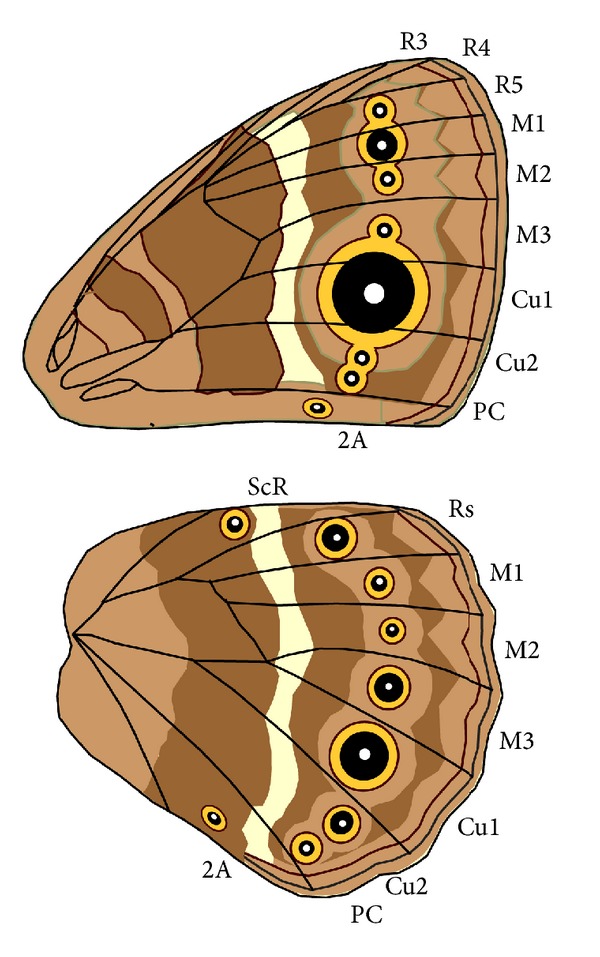
Nymphalid wing compartment nomenclature. Only compartments that had eyespots across the species surveyed are shown.

We scored eyespot wing patterns from a collection of digital images previously taken from two male and two female specimens from pinned collections housed at the Peabody Museum, Yale University, the Museum or Comparative Zoology, Harvard University, and the American Museum of Natural History, New York [11]. We scored the representatives of 399 genera previously sampled for a molecular phylogeny of the Nymphalidae and 51 additional species, for a total of 450 species. Eyespots were defined as circular pattern elements on the wing margin with at least two concentric rings of colored scales or with a single color disc and a central pupil [12]. Eyespots were scored as present (1) or absent (0) for each of the wing compartments depicted in Figure 1. Polymorphisms within a sex were coded as 0.5.

We investigated two aspects of eyespot sexual dimorphism as follows.

1. For a rough estimate of a species dimorphism, we tallied the total number of eyespots on each wing surface, and across all surfaces for each sex, and calculated the difference in this number between sexes.
2. In order to determine if dimorphic eyespots were uniformly distributed among the wing surfaces or preferentially located on particular surfaces, female eyespot values were subtracted from male eyespot values for each wing compartment bearing eyespots within a species, and these quantities were compared across wing surfaces (Figure 1).

## 3. Results

**Figure 2:**
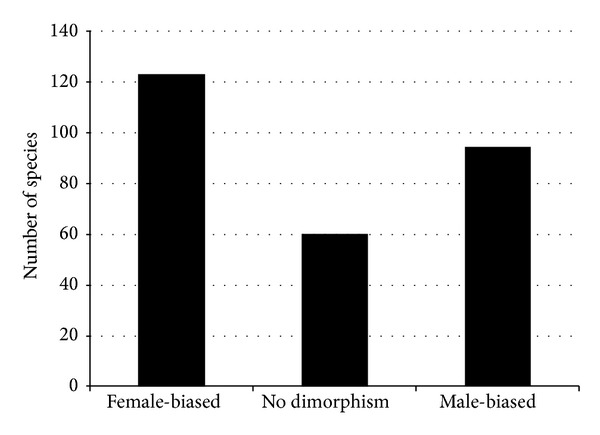
Summary statistics of total eyespot number dimorphism amongst species carrying eyespots. 123 species had more ornamented females, 94 species had more ornamented males, and 60 species had no difference in total eyespot number between males and females.

Of the 450 species surveyed for this study, 278 (61.8%) had at least one eyespot on their wings. From these, 60 species (21.6%) had no difference in total eyespot number between males and females (but they were often dimorphic in eyespot position), 123 species (44.2%) had females with more eyespots than males, and 95 species (34.2%) had males with more eyespots than females (Figure 2). In addition, the degree of eyespot dimorphism was higher for the female-biased species relative to the male-biased species. From the 123 species in which the female was more ornamented, females averaged 2.32 more eyespots than males, with a median difference of 2 eyespots. On the other hand, in the 94 species that possessed more ornamented males, males averaged 1.74 more eyespots than females, with a median difference of 1 eyespot. In summary, female nymphalid species have, on average, more eyespots than males. Lastly, of the 279 species with ornamented individuals, 148 (53.0%) had polymorphic females (with variation in eyespot number), while 174 (62.4%) had polymorphic males. Further information on the descriptive statistics of this dataset can be found in (Oliver et al., in review).

**Figure 3:**
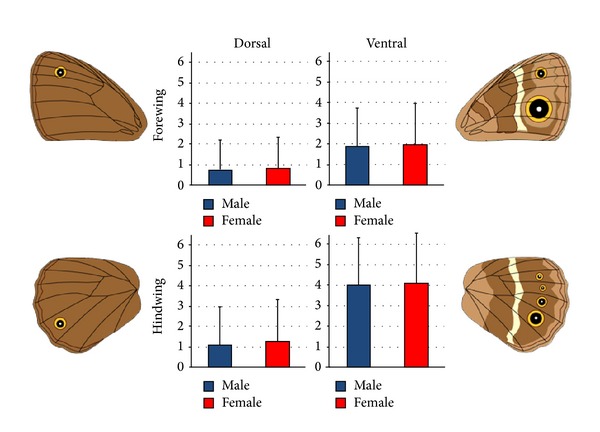
Mean number of eyespots by wing surface for males and females for all eyespot-bearing species. Females averaged slightly more eyespots than males for all wing surfaces. Dorsal surfaces had fewer eyespots than ventral surfaces and forewings fewer eyespots than hindwings. Error bars represent one standard deviation.

Eyespots do not occur in equal numbers across all wing surfaces. In both sexes, ventral surfaces, on average, have more eyespots than do dorsal surfaces, and hindwings have more eyespots than do forewings (Figure 3). The surfaces with the highest number of eyespots also display the largest proportion of sexual dimorphism: 66% of all eyespot dimorphism was found on the ventral surface—31% forewing and 35% hindwing, while only 34% was found on the dorsal surface—12% forewing and 22% hindwing. Furthermore, 57% of dimorphism was on the hindwing surfaces compared to 43% on the forewing surfaces.

**Figure 4:**
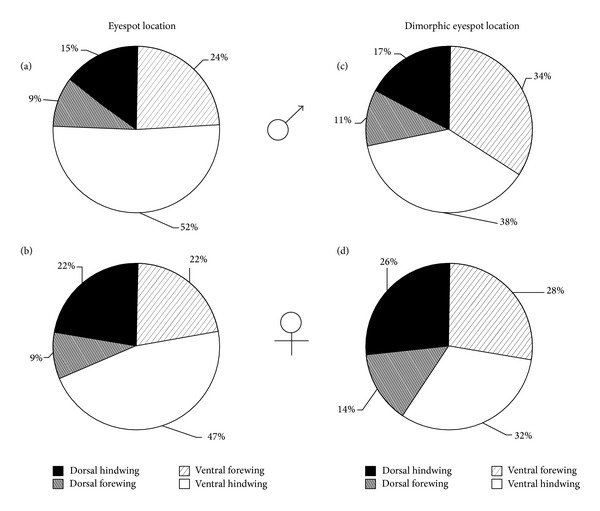
Relative distribution of total eyespot number and dimorphic eyespot number for each sex across the four wing surfaces.

Eyespots are distributed among the wing surfaces differently in males and females (Figures 4(a) and 4(b)). Ventral surfaces contained 76% of the total number of eyespots found on males but only 69% of the eyespots were found on females. Males had relatively more eyespots on their ventral hindwing when compared with females—52% to 47%. Conversely, females had relatively more eyespots on their dorsal hindwing when compared with males—22% to 15%. Males and females had an equal relative occurrence of eyespots on the ventral forewing surface—22% and 24%, respectively.

Considering only the subset of eyespots that are dimorphic, these eyespots, too, were distributed differently in males and females (Figures 4(c) and 4(d)). Males had more eyespots on their ventral surface relative to females—72% to 60%, respectively—while females had more eyespots on their dorsal surface relative to males—40% to 28%. Both males and females, however, had more eyespots on the hindwings relative to forewings (55% for males and 58% for females).

**Figure 5:**
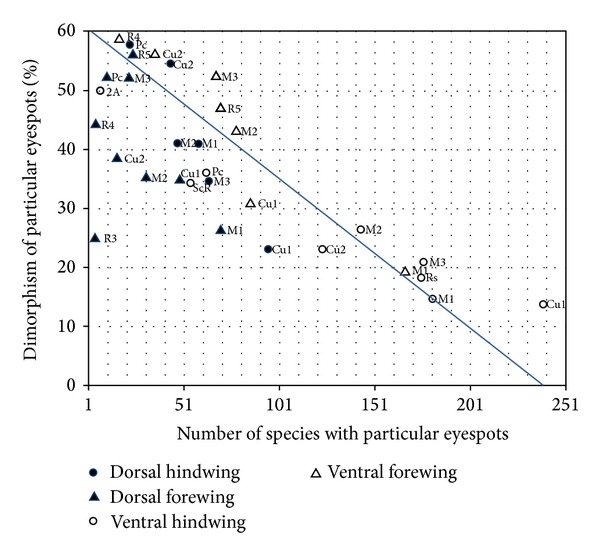
Wing compartments commonly displaying eyespots are proportionally the least dimorphic. Compartment name labels are adjacent to each point. Best fit line from Pierson correlation for all eyespots is depicted in blue.

In general, the wing compartments that most commonly contained eyespots were proportionately the least sexually dimorphic (Pearson correlation, *r* = −0.73, *P* < 0.001) (Figure 5). All surfaces, except the dorsal forewing, showed a significant negative correlation between eyespot frequency and eyespot dimorphism (Table 1). Eyespots were commonly found in four wing compartments on the ventral hindwing: the Cu1 (239 species; 86.28% of species with at least one eyespot), M1 (181 species; 65.34%), Rs (175 species; 63.18%), and M3 (176.5 species; 63.72%), and the M1 compartment on the ventral forewing (167 species; 60.29%). However, these five most common eyespot locations were also the five least dimorphic. The most common eyespot location, the Cu1 compartment on the ventral hindwing, was dimorphic in only 13.81% of the species. The second most common eyespot location, the M1 compartment of the ventral forewing, was dimorphic in only 19.46% of the species, demonstrating the tendency for common eyespot compartments to have a relatively low dimorphic rate. In contrast, the 2A and R3 compartments on the ventral forewing were proportionately the most sexually dimorphic (100% and 94.44%, resp.), but only a small proportion of species carried eyespots at these positions (0.36% and 3.25% of all eyespot-bearing species, resp.).

## 4. Discussion 

A majority of nymphalid species has females with more eyespots than males, supporting data previously obtained for the genus *Bicyclus* [10]. However, a significant proportion of species display the opposite pattern, and many species are monomorphic in total eyespot number. Ventral hindwings have, on average, the greatest share of eyespots; yet this wing surface has proportionately the least amount of sexual dimorphism relative to the other three wing surfaces. Sexual dimorphism is, thus, primarily found in wing surfaces that can be hidden from predators when the butterfly sits with its wings closed.

These results are congruent with the sexual dimorphism displayed in *B. anynana* and with both mate choice and predation experiments previously performed on this species. These results also support the comparative work performed across the genus *Bicyclus.* Both sexes of *B. anynana* are polymorphic regarding the number of eyespots on the dorsal hindwing but females have, on average, one additional eyespot relative to males (Westerman et al., pers. comm.). It is currently unclear why females have additional eyespots on this surface, but sexual selection via male choice is a possibility. Males alone notice the number of eyespots on the dorsal hindwings of females in mate choice trials, whereas females do not discriminate males based on this trait (Westerman et al., pers. comm.). Both males and females, however, notice the number of eyespots on the dorsal forewings of the opposite sex [7, 8, 13]. A more limited set of experiments showed that females do not pay attention to eyespots on the ventral surfaces of males [8], but the reciprocal experiment with males has yet to be done. A larger number of eyespots on the dorsal hindwing of females may, thus, result from sexual selection on females by males.

The ventral eyespots of butterflies are generally the most visible to natural enemies as they are displayed when butterflies rest with their wings folded over their bodies. In some circumstances (either using naïve predators or under low light conditions), the attacks tend to target the area of the eyespots, in particular the hindwings [14], allowing the butterfly to escape with parts of the wing missing. These experiments suggest that sexual dimorphism on these wing surfaces may be maladaptive because both sexes benefit equally from the predator evading mechanism provided by these eyespots. These experiments lend support to the results of the survey that show that ventral surfaces, and especially ventral hindwing surfaces, are proportionately the least sexually dimorphic. While large dorsal forewing eyespots also function as an intimidating defense in some species [15–20], this strategy may be restricted to fewer lineages.

Comparative work performed across the genus *Bicyclus *also supports the results of this survey; namely, hindwing and ventral eyespots are proportionately less sexually dimorphic than forewing or dorsal eyespots. Estimates of rates of eyespot gains and losses calculated separately for males and females indicated that eyespots on the dorsal surface evolve at higher rates and at sex-specific rates, relative to eyespots on the ventral surface [9]. This increased lability of the dorsal surface is associated with a higher frequency of dimorphic eyespots on this surface. Eyespots on *Bicyclus* forewings were also more labile and also had higher sex-specific rates of evolution relative to hindwing eyespots [9]. So, research on *Bicyclus *may explain many of the prevalent patterns in the general nymphalid dataset.

This survey showed that the most common eyespot locations were proportionately the least dimorphic. These results are interesting when contrasted against two recent studies that inferred the wing surface where eyespot first originated within nymphalids. Four of the five most common eyespots were estimated to be the original eyespots that appeared on the ventral hindwing of an ancestral nymphalid roughly 90 million years ago [11] (Oliver et al. in review). The fact that these eyespots are the most prevalent in species today may simply be due to their retention in a majority of lineages since their origin. The more recent dorsal eyespots seem to have originated 30 million years later (Oliver et al. in review). Despite their late origins, these dorsal eyespots have evolved some of the highest levels of sexual dimorphism seen across nymphalid eyespots.

Sexual differences in behavior, including basking propensity, mate searching, egg laying, courtship behavior, and associated observational angles towards the wings of the opposite sex, may all contribute to the variation in sexual dimorphisms in eyespots across wing surfaces. For example, *Bicyclus* species do not bask, so their dorsal wing patterns may not be as visible to predators and be subject to as much natural selection as the dorsal wing pattern of species that bask or those where one sex basks more than the other. In addition, when courting, *Bicyclus* males and females (females also court males in this species) both approach the opposite sex at an angle that makes their dorsal wing surfaces especially visible. Behavioral data such as this can help determine the relative importance of certain wing surfaces in mate signaling. In addition, sex-specific patterns of natural selection and further behavioral information for multiple nymphalid species are needed if we want to understand the full extent of the patterns of sexual dimorphism in this clade of butterflies.

## Figures and Tables

**Table 1:** Pearson correlation coefficient (*r*) and significance (*P*) of the correlation between eyespot frequency and eyespot dimorphism for all or for specific wing surfaces.

Surface	*r*	*P*
Eyespots from all surfaces	−0.73	<0.001***
Dorsal forewing	−0.34	0.312
Dorsal hindwing	−0.92	0.001**
Ventral forewing	−0.89	<0.001***
Ventral hindwing	−0.96	<0.001***

**P < 0.01; ***P < 0.001.
