# Corrigendum to “A Survey of Eyespot Sexual Dimorphism across Nymphalid Butterflies”

**DOI:** 10.1155/2017/2704640

**Published:** 2017-07-27

**Authors:** Christopher K. Tokita, Jeffrey C. Oliver, Antónia Monteiro

**Affiliations:** ^1^Yale College, New Haven, CT 06511, USA; ^2^Department of Ecology and Evolutionary Biology, Yale University, New Haven, CT 06511, USA; ^3^Department of Zoology, Oregon State University, Corvallis, OR 97331, USA; ^4^Department of Biological Sciences, National University of Singapore and Yale-NUS College, Singapore 117543

In the article titled “A Survey of Eyespot Sexual Dimorphism across Nymphalid Butterflies” [1], there was a missing affiliation for the first author. The corrected authors' list and affiliations are shown above.
